# Identification of QTLs for Grain Protein Content in Russian Spring Wheat Varieties

**DOI:** 10.3390/plants11030437

**Published:** 2022-02-05

**Authors:** Irina N. Leonova, Antonina A. Kiseleva, Alina A. Berezhnaya, Anatoly I. Stasyuk, Ivan E. Likhenko, Elena A. Salina

**Affiliations:** 1 The Federal Research Center Institute of Cytology and Genetics, Siberian Branch of the Russian Academy of Sciences (ICG SB RAS), Prospekt Lavrentyeva 10, 630090 Novosibirsk, Russia; antkiseleva@bionet.nsc.ru (A.A.K.); al.berezhnaya1@gmail.com (A.A.B.); stasyuk@bionet.nsc.ru (A.I.S.); salina@bionet.nsc.ru (E.A.S.); 2 Siberian Research Institute of Plant Production and Breeding—Branch of the Institute of Cytology and Genetics, Siberian Branch of the Russian Academy of Sciences, 630501 Krasnoobsk, Russia; likhenko@mail.ru

**Keywords:** bread wheat, grain protein content, *NAM-A1* gene, association mapping

## Abstract

Most modern breeding programs aim to develop wheat (*T. aestivum* L.) varieties with a high grain protein content (GPC) due to its greater milling and cooking quality, and improved grain price. Here, we used a genome-wide association study (GWAS) to map single nucleotide polymorphisms (SNPs) associated with GPC in 93 spring bread wheat varieties developed by eight Russian Breeding Centers. The varieties were evaluated for GPC, grain weight per spike (GWS), and thousand-kernel weight (TKW) at six environments, and genotyped with 9351 polymorphic SNPs and two SNPs associated with the *NAM-A1* gene. GPC varied from 9.8 to 20.0%, depending on the genotype and environment. Nearly 52% of the genotypes had a GPC > 14.5%, which is the threshold value for entry into high-class wheat varieties. Broad-sense heritability for GPC was moderate (0.42), which is due to the significant effect of environment and genotype × environment interactions. GWAS performed on mean GPC evaluated across six environments identified eleven significant marker-trait associations, of which nine were physically mapped on chromosome 6A. Screening of wheat varieties for allelic variants of the *NAM-A1* gene indicated that 60% of the varieties contained the *NAM-A1c* allele, followed by 33% for *NAM-A1d*, and 5% for *NAM-A1a* alleles. Varieties with the *NAM-A1d* allele showed significantly (*p* < 0.01) smaller GPC than those with *NAM-A1c* and *NAM-A1a*. However, no significant differences between *NAM-A1* alleles were observed for both GWS and TKW.

## 1. Introduction

Wheat (*Triticum aestivum* L.) plays a key role in most countries worldwide, as a major source of plant protein. According to the FAO data, the area of the worldwide arable land occupied by wheat varieties in recent years is 219 million hectares, of which five major producers and exporters of wheat (China, India, Russia, USA, and Canada) account for 50% of the total area (https://www.fao.org/faostat/ (accessed on 28 December 2021)). Although worldwide annual wheat production has increased over the years, reaching 761 million tons in 2021, the demand for wheat has also constantly increased [1]. The Russian Federation is one of the largest producers and exporters of common wheat. The sown area occupied by winter and spring wheat is constantly increasing, and amounts to about 29 million hectares [2]. Over the past two decades, there has been a significant increase in the yield of winter and spring wheat varieties due to the development and introduction of new high-yielding wheat cultivars [3]. In addition to grain yield, however, modern wheat varieties are expected to consist of desirable allele combinations for multiple traits, including high grain protein content (GPC), early maturity, short plant height, user-specific end-use quality traits, and resistance to major diseases. GPC is important due to its direct effect on milling and baking quality, nutritional value, and grain price. Wheat varieties with a high GPC and good baking qualities have a higher market value.

Bread-making qualities are complex traits that depend on the total GPC, the composition of storage proteins, and on endosperm structure or grain hardness [4,5,6]. Wheat quality is conditioned by a large number of genes and quantitative trait loci (QTLs), which are significantly influenced by environmental factors [7,8]. Various investigations have demonstrated that the heritability of GPC is moderate, and the environment is a key factor determining the phenotypic expression of the trait [9,10,11]. Genetic mapping studies performed using different types of populations identified QTLs associated with GPC in almost all chromosomes of tetra- and hexaploid wheat [12,13,14]. Prasad et al. [9], for example, reported 13 QTLs on chromosomes 2A, 2B, 2D, 3D, 4A, 6B, 7A, and 7D that individually explained phenotypic variance from 3 to 32%. In another study, nine QTLs on chromosomes 1B, 3B, 4A, 5A, 5B, 6B, 7A, 7B, and 7D were identified in a recombinant inbred line (RIL) population, derived from a cross between a winter wheat variety *T. aestivum* L. var. ‘Forno’ and *T. spelta* L. variety ‘Oberkulmer’ [15]. Using an association mapping panel of 196 common wheat varieties of various geographic origins, another study reported 15 genomic regions associated with GPC across nine chromosomes [16].

Of the numerous QTLs associated with GPC, *Gpc-B1* (*NAM-B1*) is the most important gene that is located on the short arm of chromosome 6B [17,18,19,20]. The wild-type allele confers high levels of GPC and micronutrients (e.g., iron, zinc) but it reduces grain yield. Although the effect of the wild-type *Gpc-B1* allele on GPC in common wheat is much less than in tetraploid wheat, this gene is characterized by a stable contribution to the phenotypic variation of the trait under various environments [21]. However, the wild-type allele has had a negative effect on other traits, including kernel size, thousand-kernel weight, and grain yield [17,22,23]. Grain weight per spike and 1000-kernel weight have been frequently used as indicators of grain yield.

The *NAM-B1* gene has been reported in a few commercial wheat cultivars. For example, screening of 196 common wheat varieties from the INRA core collection showed that only five of them carry the wild-type allele of *NAM-B1* [18]. Another study by Chen et al. [24] screened 365 winter bread wheat varieties and advanced lines cultivated in China, but the authors did not find any variety that contained a functional *NAM-B1* allele. Other *NAM* genes have been identified in hexaploid wheat, which includes *NAM-A1* and *NAM-D1* that are located on chromosomes 6A and 6D, respectively. It is assumed that *NAM-A1* performs a similar function as *NAM-B1* [25,26,27,28]. There is no information on chromosomal localization and allelic composition of loci associated with GPC in wheat varieties developed or grown in Russia. The objectives of this study were, therefore, to identify SNPs associated with GPC in Russian spring wheat varieties, and compare the phenotypic effects of *NAM-A1* gene-specific markers.

## 2. Results

### 2.1. Phenotypic Variation

**Figure 1:**
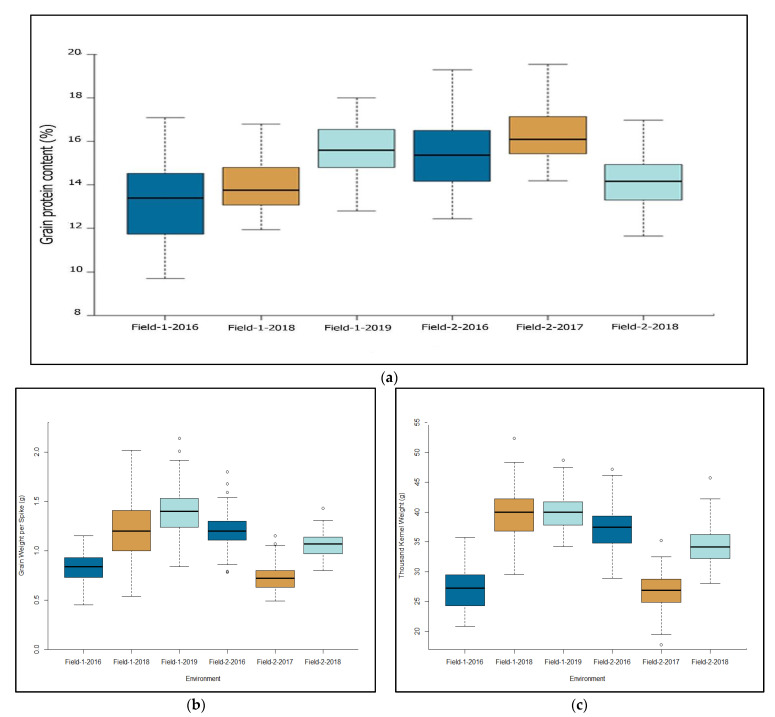
Box plot of (**a**) grain protein content, (**b**) grain weight per spike, and (**c**) thousand-kernel weight in Russian spring wheat varieties evaluated at six environments (two locations × three years).

Figure 1 summarizes the three traits recorded in individual environments. GPC, GWS, and TKW across the six environments ranged from 9.7 to 20.0%, from 0.8 g to 1.4 g, and 28.1 and 42.2 g, respectively (Figure 1a, Table S1). Most varieties (68.8%) had GPC that varied between 14% and 16%, compared with 12.9% and 18.3% that possessed a GPC greater than 16% and smaller than 14%, respectively. GPC recorded in Field-2 was significantly (*p* < 0.001) greater (15.2 ± 0.09) than in Field-1 (14.3 ± 0.1), but both TKW and GWS in Field-2 were significantly smaller than in Field-1 (Figure 1b,c).

Analysis of variance (ANOVA) showed significant differences among the 93 genotypes, six environments, and genotype × environment (*G*×*E*) interaction for GPC (Table S2). ANOVA performed on GWS and TKW data indicated significant differences among genotypes and environments, but not for *G*×*E* interactions. Broad sense heritability estimated from the data of six environments was 0.42 for GPC, 0.57 for GWS, and 0.62 for TKW. There were highly significant positive correlations between TKW and GWS (r = 0.71). GPC was negatively correlated with both GWS and TKW, but the correlation coefficients were low (r = 0.296 and 0.199, respectively).

### 2.2. Population Structure and Genome-Wide Association Study

The 93 spring wheat varieties were genotyped with the Illumina Infinium 15K array and two functional SNPs associated with the *NAM-A1* gene of which 9351 SNPs, plus the two functional SNPs, were polymorphic in the germplasm. The number of polymorphic SNPs per chromosome varied from 96 on chromosome 4D to 747 on 2B chromosome (Table 1). One hundred and thirteen of the polymorphic SNPs were not assigned into any of the chromosomes. A plot of the first three principal components (PCs) that accounted for 27.2% of the genetic variation demonstrates the existence of three groups (Figure S1a). PC analysis applied in our study is consistent with the clustering performed by Kiseleva et al. [29] using the STRUCTURE-like algorithm LEA (Figure S1b).

**Figure 2:**
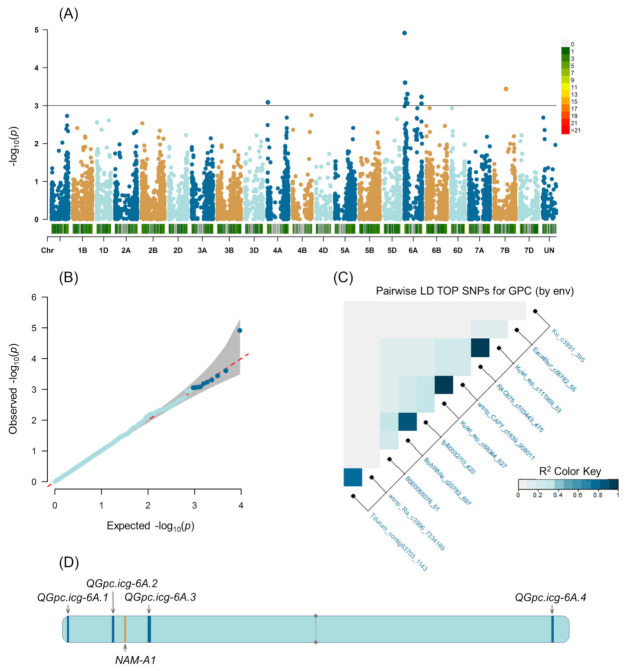
Summary of genome-wide association studies based on the grain protein content in six environments. (**A**) Manhattan plot; (**B**) quantile–quantile plot; (**C**) LD plot representing the association of significant SNP markers with the color key indicating the strength of LD from 0 to 1; (**D**) schematic illustration of the localization of QTLs and the *NAM-A1* locus on chromosome 6A with the vertical line in the middle referring to the centromere position.

GWAS performed using the mixed linear model (MLM) and GPC data of individual environments showed significant (*p*-value < 0.001) marker-trait associations (MTAs) for 50 SNPs (Table S3, Figure S2). Using the IWGSC RefSeq v1.0 physical information [30,31], forty-nine of the significant SNPs were distributed across ten chromosomes (1B, 2B, 3A, 3B, 4A, 5B, 5D, 6A, 6B, and 6D). It should be noted that three SNPs were significantly associated with the GPC recorded in three environments: wsnp_Ra_c3996_7334169 in Field-2-2017, Field-2-2018, and Field-1-2019; Tdurum_contig63703_1143 in Field-2-2017 and Field-1-2019, and BS00065076 in Field-2-2018 and Field-1-2019 (Table S3). Twenty-one of the significant SNPs (42%), however, were located on chromosome 6A. GWAS, performed on the best linear unbiased estimations (BLUEs) across six environments, identified eleven SNPs associated with GPC, of which nine were localized physically [30] and genetically [32] on chromosome 6A (Table 2, Figure 2A). One of the significant SNPs (Ku_c3891_395) was localized on chromosome 4A, and another SNP (Excalibur_c55782_55) was not mapped (Table 2). Each significant SNP explained between 19.3% and 27.7% with an overall average of 24.7%. The significant SNPs identified on chromosome 6A were located at 6.7 Mb (*QGpc.icg-6A.1*), 23.4 Mb, 61.0–61.2 Mb (*QGpc.icg-6A.2*), 103.7–107.1 Mb (*QGpc.icg-6A.3*), and 569.9–597.0 Mb (*QGpc.icg-6A.4*). Candidate gene search using the physical interval of the four QTLs summarized in Table 2 identified a total of 1 gene for *QGpc.icg-6A.1*, 5 genes for *QGpc.icg-6A.2*, 38 genes for *QGpc.icg-6A.3*, and 2 genes for *QGpc.icg-6A.4* (Table S4).

An analysis of linkage disequilibrium (LD) (Figure 2C) of markers significantly associated with GPC grouped the SNPs on chromosome 6A into four regions that correspond to *QGpc.icg-6A.1* (wsnp_Ra_c3996_7334169, Tdurum_contig63703_1143), *QGpc.icg-6A.2* (Kukri_rep_c68344_627, wsnp_CAP7_c1839_908011), *QGpc.icg-6A.3* (BobWhite_c20782_697, tplb0032i10_420), and *QGpc.icg-6A.4* (RAC875_c103443_475, Kukri_rep_c111369_53) (Table 2, Figure 2D). A positive additive phenotypic effect (1.7–3.8%) was shown for the markers Kukri_rep_c68344_627, wsnp_CAP7_c1839_908011, tplb0032i10_420, and BobWhite_c20782_697 that were physically located on the short arm of chromosome 6A. Localization of these SNPs coincides with the position of the *NAM-A1* locus, according to the physical [30] map of chromosome 6A (Table 2, Figure 2D).

### 2.3. Allelic Polymorphism of NAM-A1 Gene

**Figure 3:**
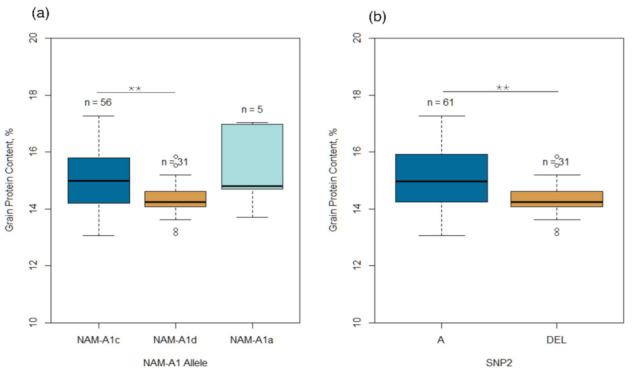
Variability of grain protein content in 93 spring wheat varieties based on (**a**) the allelic composition of three *NAM-A1* haplotypes, and (**b**) polymorphism based on the SNP2. Asterisks (**) indicate statistically significant differences (*p* < 0.01) between *NAM-A1* alleles. The horizontal line inside the box is the median.

Since most MTAs were found on chromosome 6A, we screened the 93 wheat genotypes using two SNPs (SNP1 and SNP2) that were previously reported to detect the allelic composition of the *NAM-A1* gene [33]. SNP1 is located in the second exon (6AS: 4397602_16233) and carries the C/T polymorphism followed by the substitution of alanine for valine in the protein sequence. SNP2 is located at the end of the coding sequence (exon 3, 6AS: 4397602_17020) and corresponds to the A/deletion polymorphism leading to a shift in the reading frame. Both types of polymorphism were found in the present study (Figure 3, Table S5). Under the nomenclature proposed by Cormier et al. [33], the alleles amplified by both SNP1 and SNP2 were designated as *NAM-A1a*, *NAM-A1c*, and *NAM-A1d*, which were observed in 5.3%, 60.2%, and 33.3% of the 93 varieties, respectively (Table S5). The *NAM-A1a* allele was rare, while *NAM-A1c* was the most abundant. The *NAM-A1* allelic composition showed any pattern that was neither on the clustering of the collection into separate groups nor the breeding programs that developed the varieties.

For SNP2, a deletion was detected in 33.3% of the varieties, while nucleotide “A” was identified in 65.6% of the varieties (Table S5). The *NAM-A1b* allele was not found. Based on the comparative analysis of GPC in varieties containing different allelic variants of the *NAM-A1* gene, genotypes with the *NAM-A1d* allele showed a significantly (*p* < 0.01) smaller GPC than those with *NAM-A1a* and *NAM-A1c* (Figure 3). On the other hand, no significant differences in GWS and TKW were observed among the three *NAM-A1* alleles.

## 3. Discussion

### 3.1. Phenotypic Variation and MTAs

This study evaluated diverse Russian spring wheat varieties under six environments. According to the standards that determine the commodity classification of wheat in the Russian Federation, GPC for high-class wheat varieties should be at least 14.5%, which was observed in about 52% of the varieties used in the current study (Figure 1A, Table S1). A moderate broad-sense heritability for GPC was observed (0.42) due to the significant effect of environments and G×E interactions, which agrees with several previous studies conducted on tetraploid and hexaploid wheat [34,35,36,37].

Using GWAS, we identified 50 SNPs across ten chromosomes (1B, 2B, 3A, 3B, 4A, 5B, 5D, 6A, 6B, 6D) that were significantly associated with the GPC recorded in each environment (Table S3). Each significant SNP explained between 6.1–41.7% of the phenotypic variation. Several previous studies reported multiple QTLs for GPC on chromosomes 1A, 1B, 2A, 2B, 3A, 4A, 5B, 6A, 7A, and 7B [17,37,38,39,40]. Our GWAS performed on the GPC data averaged across all six environments; however, we identified only eleven significant SNPs, nine of which were located on chromosome 6A (Table 2), which agrees with several previous studies. Using 486 modern wheat cultivars, landraces, and breeding lines from China, America, and Europe, Lou et al. [41] reported 10 QTLs associated with GPC. One of the QTLs for GPC (*QNGpc.cau-6A*) consisted of a cluster of 30 SNPs between 63 and 83 Mb on chromosome 6A that explained 2.7–4.8% of phenotypic variation. In the present study, two of the eleven SNPs associated with GPC fell within this range *QGpc.icg-6A.2* (Table 2). It should also be noted that: (1) *QGpc.icg-6A.2* accounted for 24.6–26.8% of the phenotypic variance for GPC, which is over five-fold greater than the effect reported by Lou et al. [41]; and (2) the *NAM-A1* gene has been physically located at ~77.1 Mb on chromosome 6A [30]. In another study, Muqaddasi et al. [37] reported SNPs significantly associated with GPC on chromosome 6A in a panel of winter bread wheat varieties of European origin. However, one of the SNPs (Tdurum_contig46828_730) is located at ~643.2 Mb on the IWGSC RefSeq v1.0 map, which is about 46 Mb away from one of the QTLs (*QGpc.icg-6A.4*) identified in the present study (Table 2). Our results, together with others, suggest the presence of two or more genomic regions associated with GPC on chromosome 6A.

Candidate gene search using the physical interval of the four QTLs summarized in Table 2 identified a total of forty-six *T. aestivum* genes (Table S4). *TraesCS6A02G132700* (6A:104531253-104533070) is one of the candidate genes that encodes a CBS-containing protein, which is expressed predominantly in the developing grain; this gene is slightly or not at all expressed in the other plant tissues [42]. Since proteins with a CBS domain can have a regulatory function, the *TraesCS6A02G132700* gene most likely plays a role in grain protein content. In addition to chromosome 6A, we also detected a significant SNP on the short arm of 4A (Ku_c3891_395). According to several studies, chromosome 4A may contain GPC-associated loci, although most QTLs were reported on the long arm of this chromosome [9,21,39].

The power of QTL detection depends on several factors, including population size, marker density, population structure, the genetic architecture of the trait, and trait heritability [43,44]. Our population size was 93 spring wheat varieties and advanced breeding lines, which is comparable to some previous studies [45,46,47]. However, the small size may have restricted our ability in detecting more significant MTAs and/or biased the effect of each identified SNP, which varied between 18.7% and 27.7% (Table 2).

### 3.2. Effect of NAM-A1 Gene on GPC and Yield Components

The proportion of *NAM-A1a*, *NAM-A1c*, and *NAM-A1d* haplotypes in the Russian spring wheat was 5.3%, 60.2%, and 33.3%, respectively (Table S5). It should be noted that the allelic diversity of the *NAM-A1* gene in Russian varieties was correlated neither with the breeding programs that developed the varieties nor with the heading date [48,49]. Cormier et al. [33] studied the allelic variation of the *NAM-A1* gene in 367 wheat varieties of different geographical origins, and reported the predominant presence of the *NAM-A1c* (T/A) allele. At the same time, 72% of the elite European varieties had a *NAM-A1d* (T/Del) allele as compared with just 0.3% for the *NAM-A1b*. Among 51 wheat varieties of the Australian selection, *NAM-A1a* was most common, but *NAM-A1b* was detected only in one variety [27]. The data of Alhabbar et al. [50,51] confirmed the results of a previous study on the predominant presence of the *NAM-A1a* allele in the genome of early maturing Australian varieties. On the contrary, the *NAM-A1d* and *NAM-A1b* were common in the Pakistan wheat varieties [52].

Orlovskaya et al. [53] studied introgression lines derived from crosses involving wheat varieties Saratovskaya 29, Rassvet, Festivalnaya, Chinese Spring, Belorusskaya 80, and Pitic S62 with wild relatives (*T. durum*, *T. dicoccum*, *T. dicoccoides*, *T. timopheevii*). Four out of six wheat varieties contained *NAM-A1d*. Among the wild relatives, the most common haplotype was *NAM-A1c*; *NAM-A1b* was not identified among the studied genotypes. Combined across all six environments, we found higher mean GPC among varieties with the *NAM-A1a* than both *NAM-A1c* and *NAM-A1d*, which was consistent with the data of Cormier et al. [33]. Studies by Alhabbar et al. [50,51], on the contrary, reported lower GPC in *NAM-A1a* haplotypes and higher GPC for non-*NAM-A1a* haplotypes.

## 4. Materials and Methods

### 4.1. Plant Materials and Phenotyping

Plant material included 93 spring wheat varieties and advanced breeding lines (hereafter referred to as varieties) developed by eight Russian Breeding Centers and adapted for cultivation in the Siberian region of the Russian Federation. The list of varieties and their origin is presented in Table S1. More detailed information about the pedigree of the varieties can be found in GRIS Internet resources [54]. Seeds were maintained and multiplied in the Federal Research Center Institute of Cytology and Genetics SB RAS (ICG SB RAS, Novosibirsk). The wheat varieties were grown in six environments on two localities of the Western Siberian region of Russia designated as Field-1 (54.9191° N, 82.9903° E) in 2016, 2018, and 2019, and Field-2 (54.8475° N, 83.1095° E) in 2016, 2017, and 2018. Varieties were sown in mid-May and harvested in September of every year in two replicates on a plot of 1 m wide, with 60 grains per row, at a spacing of 25 cm between rows. The soils at Field-1 and Field-2 were leached chernozem and gray forest, respectively.

Grain protein content was determined using infrared express analyzer OmegAnalyzer G (Bruins Instruments, Munich, Germany) using 10 g seeds in triplicate according to the manufacturer’s instructions. The mean grain weight per spike and the 1000-kernel weight were calculated from 25 plants of each variety.

In 2016, the weather was dry with monthly temperatures in May, June, and July, on average, 4 °C higher than the long-term values. The total precipitation (140 mm) that fell during the heading and grain-filling period in 2016 was almost 40% smaller than the long-term average values (220.0 mm). Total precipitation during the growing season of 2017 was 271.0 mm, which is 10% more than long-term average values (220.0 mm). In 2019, the amount of precipitation (205.0 mm) was comparable to the long-term. The average monthly air temperature in 2017 and 2019 did not differ from the average long-term values. The growing season of 2018 was characterized by low temperatures in May (on average, 5 °C below normal) and high waterlogging in May–June, compared with other seasons. Therefore, the most favorable weather for yield was in 2017 and 2019.

### 4.2. Statistical Analysis of Phenotype Data

The phenotype data analyses were performed using R. The two-way ANOVA was performed to determine the significance of differences among the genotypes and the environments using genotypes as a fixed effect, and environments and replications as random effects. Pearson’s correlation coefficients (r) were calculated to explore the association among GPC, grain weight per spike, and thousand-kernel weight. The significance of differences between the mean values of the two sample sets was determined using the Student’s t-test. The broad-sense heritability (H^2^) across all environments was calculated as:

$$H^{2}=\frac{\sigma_{G}^{2}}{\sigma_{G}^{2}+\left(\frac{\sigma_{G\times E}^{2}}{Env}\right)+\left(\frac{\sigma_{e}^{2}}{Env\times Rep}\right)}$$

where $\sigma_{G}^{2}$, $\sigma_{G\times E}^{2}$, and $\sigma_{e}^{2}$ are the genotypic variance, *G*×*E* interaction, and residual (error) variance, respectively, while *Env* and *Rep* are the number of environments and the number of replicates within each environment, respectively.

### 4.3. DNA Isolation, Genotyping, and Genome-Wide Association Study

Genomic DNA was isolated from 5–7-day-old seedlings using a modified sodium bisulfite protocol, as described by Kiseleva et al. [55]. DNA purification for SNP genotyping was performed using the “Bio-Silica” kit according to the manufacturer’s protocol. The DNA concentration was measured with a NanoDrop M2000 (Thermo Scientific, Waltham, MA, USA). Genotyping was carried out with the Illumina Infinium 15K array at TraitGenetics company (Gatersleben, Germany, www.traitgenetics.com (accessed on 28 December 2021)) that consisted of 13,006 SNP markers that were mapped in the wheat genome [30,32]. SNP markers with a minor allele frequency (MAF) less than 5% and missing data > 5% were excluded from the genotype data set. The International Wheat Genome Sequencing Consortium (IWGSC) RefSeq v. 1.0 chromosome location and position of each SNP was obtained from the Triticeae Toolbox [30], while the consensus genetic map was obtained from Wang et al. [32]. A genome-wide association study (GWAS) was performed using the mixed linear model (MLM) method implemented in the TASSEL v. 5.2.50 software [56] on the following data: (a) the 9351 polymorphic SNPs, (b) the first three principal components to account for population structure (Q-matrix), (c) kinship (K-matrix), and (d) the best linear unbiased estimations (BLUEs) computed within each environment and combined across six environments. Both the principal component (PC) and kinship matrix were computed from the 9351 polymorphic SNPs using TASSEL v5.2.50 [56].

Quantile–quantile and Manhattan plots were generated using the R package “GWASTools” [57]. Significant marker-trait associations were declared at *p*-values < 0.001 after adjustment for multiple testing using the Benjamini–Hochberg method [58]. Linkage disequilibrium (LD) between SNP markers was calculated using the R package “genetics” [59]. LD decay plots were generated using the R package “LDheatmap” [60]. Chromosome positions of SNP markers were established according to the IWGSC 1.0 annotation [30] and consensus genetic maps [32]. The start and end physical positions of each QTL were used to search for candidate genes at the Ensemble Plants using *Triticum aestivum* genome (https://plants.ensembl.org/index.html (accessed on 28 December 2021)).

### 4.4. Genotyping with NAM-A1 Specific SNPs

A pair of previously reported primers, NAMA1F (5′-TAGCTAGCTTGCTAGGGGGAAC-3′) and NAMA1R (5′-CAACTACTGGCTACACTTGCAAA-3′) [27], were used for amplification of the full-length *NAM-A1* gene sequences. The PCR products were separated on 2.0% agarose gel, the target bands were excised from the gel and purified using QIAquick^®^ Gel Extraction Kit (QIAGEN, Germantown, MD, USA). SNP detection was performed by the Sanger sequencing method. PCR products were directly sequenced using a BigDye Terminator v. 3.1 kit (Applied Biosystems, Waltham, MA, USA) with primers 512-R (5′-TGCTAGCTATACCGTGCGAT-3′) and 1191-F (5′-GGACGTACCATCAACACCAT-3′) reported by Orlovskaya et al. [53] to detect SNP1 and SNP2 polymorphisms in *NAM-A1* gene, respectively. Sequencing products were analyzed at the Collective Use Center “Genomika” of the SB RAS.

## 5. Conclusions

Most of the Russian spring wheat varieties had a grain protein content greater than the 14.5% for entry into high-class wheat varieties (also “strong” or “valuable” class). Genome-wide association analysis conducted on the mean GPC of the six environments identified significant MTAs on chromosomes 4A and 6A, but most SNPs were mapped at five regions on 6A. Each significant SNP explained between 18.7% and 27.7% of the GPC averaged across the six environments, which may have been upward biased to the relatively small population size. Haplotype screening of the *NAM-A1* gene using two SNPs showed that 95% of varieties contained either the *NAM-A1c* (60%) or *NAM-A1d* (33%) alleles, with the *NAM-A1a* allele accounting for the remaining 5%. The results presented here provide researchers with valuable data for selecting parental lines for new breeding starts, and understanding the SNPs that map close to the *NAM-A1* gene. However, the physical information (chromosomes and their positions) presented in this paper is based on IWGSC RefSeq v1.0, which may be different from the latest version (IWGSC RefSeq v2.1).

## Figures and Tables

**Table 1:** Summary of the polymorphic genome-wide SNPs used in the present study across three wheat genomes.

Genome	Chromosome							Unknown	Total
	1	2	3	4	5	6	7		
A	571	499	479	382	514	532	570	-	3547
B	567	747	535	215	681	600	463	-	3808
D	347	429	213	96	288	296	214	-	1883
Unknown	-	-	-	-	-	-	-	113	9351

**Table 2:** SNP markers associated with grain protein content (GPC) in 93 spring wheat varieties evaluated across six environments.

Marker	Chromosome Based on IWGSC Refseq v. 1.0	Position		F *	*p*-Value	Favorable Allele	R^2^ **	QTL
		IWGSC Refseq v. 1.0 (bp)	90K Array Consensus Map, cM					
Tdurum_contig63703_1143	6A	6733494	16.58	9.75	0.00018	T	19.3	*QGpc.icg-6A.1*
wsnp_Ra_c3996_7334169	6A	6733803	16.95	22.11	0.00001	A	21.8	
BS00065076_51	6A	23372012	43.09	9.16	0.00030	A	27.1	
Kukri_rep_c68344_627	6A	61037678	71.23	7.67	0.00095	T	26.8	*QGpc.icg-6A.2*
wsnp_CAP7_c1839_908011	6A	61212495	71.23	12.79	0.00063	A	24.6	
tplb0032i10_420	6A	103760106	76.92	7.59	0.00100	A	23.6	*QGpc.icg-6A.3*
BobWhite_c20782_697	6A	107113198	77.13	8.59	0.00044	G	22.8	
RAC875_c103443_475	6A	596903177	125.22	7.89	0.00078	A	27.7	*QGpc.icg-6A.4*
Kukri_rep_c111369_53	6A	597031623	125.22	8.34	0.00054	T	27.7	
Ku_c3891_395	4A	27673099	37.05	12.19	0.00083	C	25.4	
Excalibur_c55782_55	Unknown	5950	127.65 (7D)	8.54	0.00047	G	18.7	

*—F-test for marker; **—marker R^2^ (%) for grain protein content.

## Data Availability

The data presented in this study are available in Supplementary Materials.

## Supplementary Materials

The following supporting information can be downloaded at: https://www.mdpi.com/article/10.3390/plants11030437/s1, Figure S1: Population structure among 93 spring wheat varieties. Figure S2: Manhattan and QQ plots o based on genome-wide association mapping of grain protein content recorded in 93 spring wheat varieties at six location x year combinations (environments). Table S1: Summary of the grain protein content (%) data recorded in 93 spring wheat varieties evaluated between 2016 and 2019 at Field-1 and Field-2 (six environments in total). Table S2: Analysis of variance for grain protein content (GPC), grain weight per spike (GWS) and thousand kernel weight (TKW) in the 93 spring wheat genotypes. Table S3: List of SNP markers associated with grain protein content in six environments. Table S4: Candidate gene list. Table S5: Allelic polymorphism of *NAM-A1* gene in spring wheat varieties.
