# Supplementary material for: Identification of QTLs for Grain Protein Content in Russian Spring Wheat Varieties
Source: Plants (Basel). 2022 Feb 5;11(3):437. doi: 10.3390/plants11030437 (PMC8840037; doi:10.3390/plants11030437)
Supplement: Supplementary file 1 [file plants-11-00437-s001.zip › Figure S1.pdf]

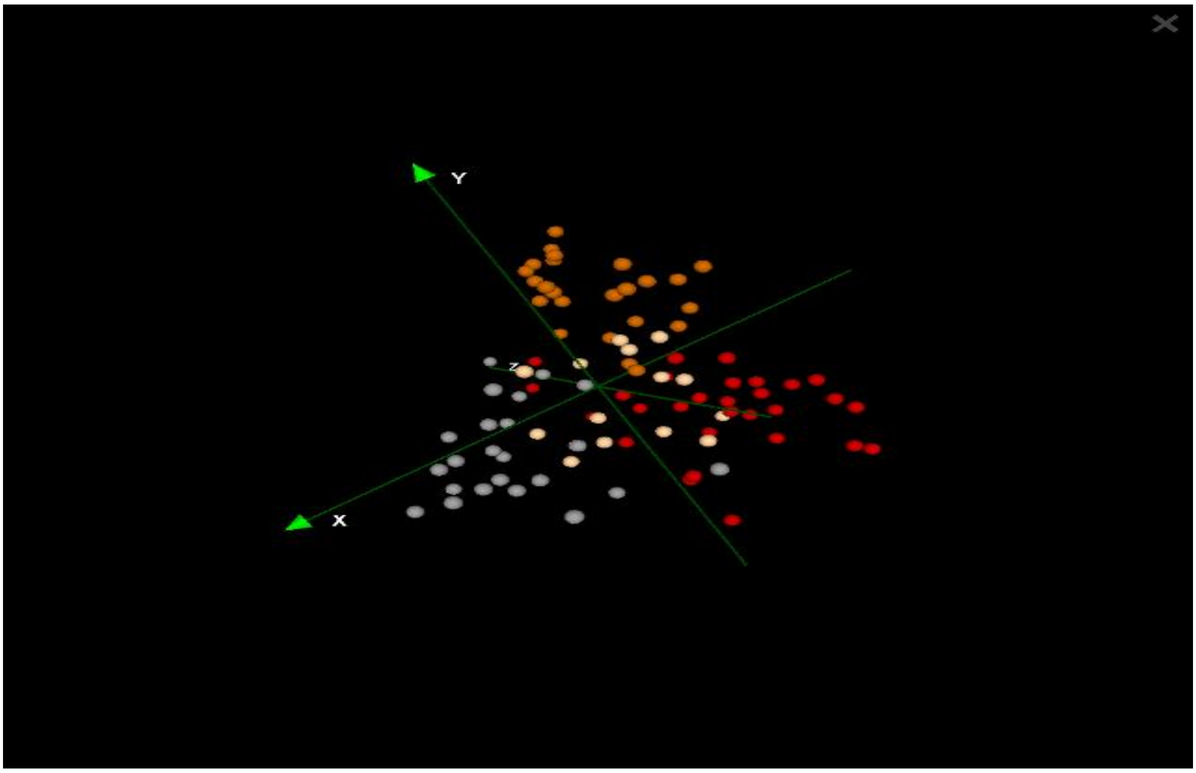

(a)

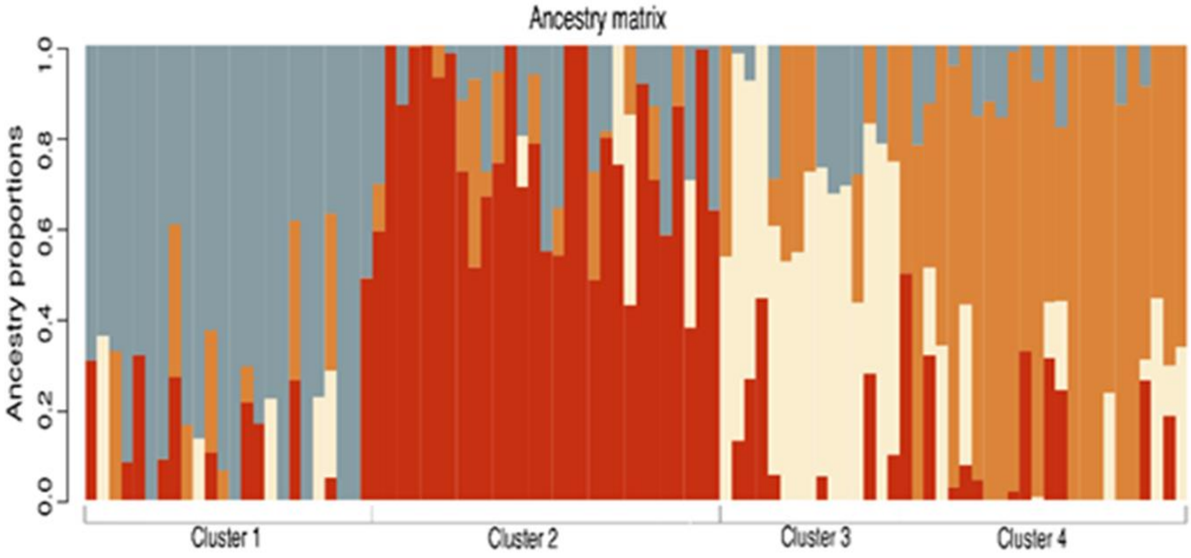

(b)

Figure S1. Population structure among 93 spring wheat varieties based on (a) a plot of PC1 (14.2%), PC2 (8.7%), and PC3 (4.3%) from principal component analysis; (b) for comparison purpose, the population structure based on STRUCTURE is provided, which has been published in Kiseleva et al. [29].
