# Supplementary material for: Identification of QTLs for Grain Protein Content in Russian Spring Wheat Varieties
Source: Plants (Basel). 2022 Feb 5;11(3):437. doi: 10.3390/plants11030437 (PMC8840037; doi:10.3390/plants11030437)
Supplement: Supplementary file 1 [file plants-11-00437-s001.zip › Figure S2.pdf]

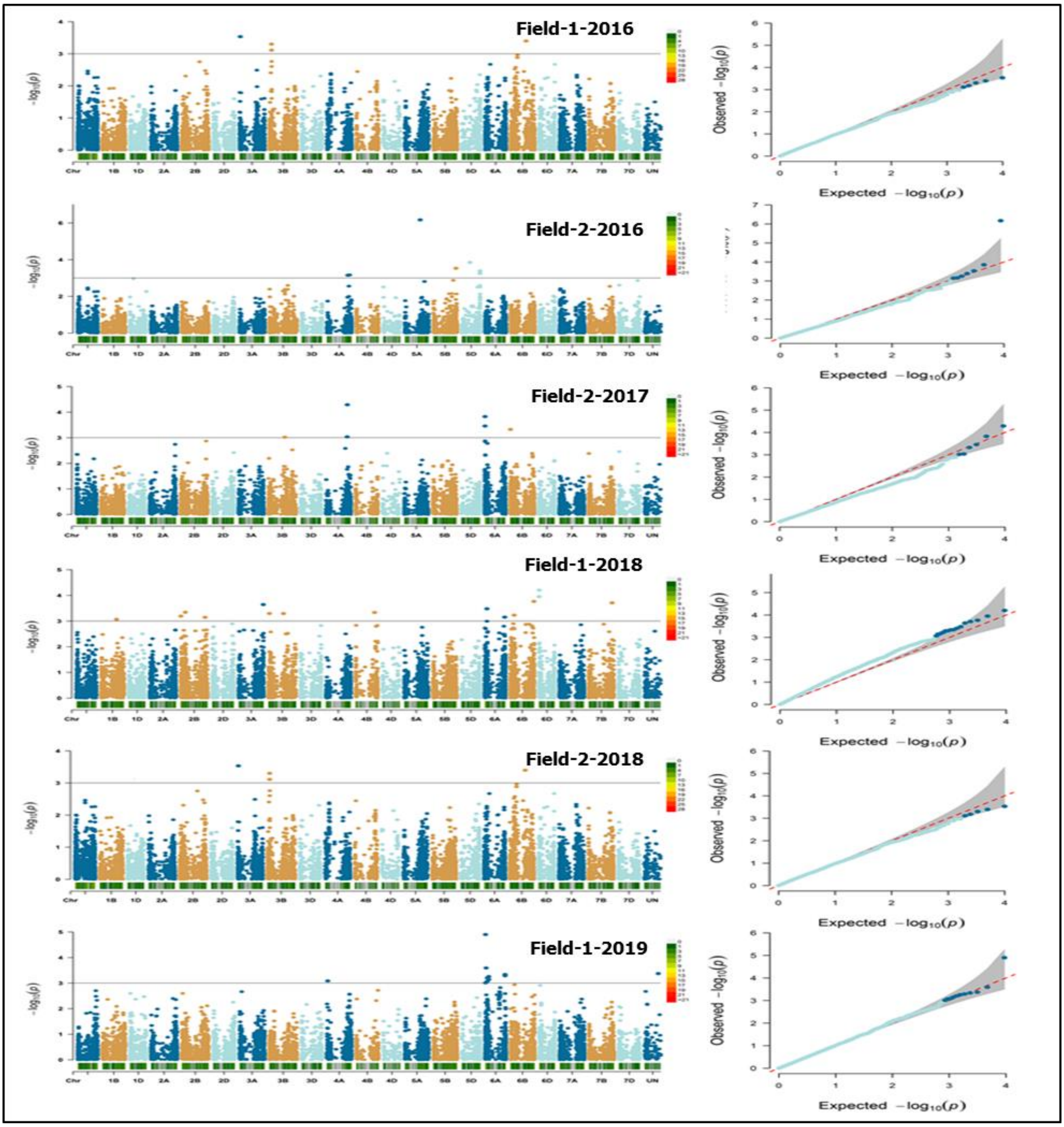

Figure S2. Manhattan and QQ-plots based on genome-wide association mapping of grain protein content recorded in 93 spring wheat varieties at six location x year combinations (environments).
